# Daily Functioning of Veterans With Type 2 Diabetes: Protocol for an Ambulatory Assessment Study

**DOI:** 10.2196/53874

**Published:** 2023-11-20

**Authors:** Jennalee S Wooldridge, Jessica L Morse, Jorge Delgado, Niloofar Afari

**Affiliations:** 1 VA San Diego Healthcare System San Diego, CA United States; 2 Department of Psychiatry University of California San Diego San Diego, CA United States; 3 VA Center of Excellence for Stress and Mental Health San Diego, CA United States

**Keywords:** type 2 diabetes mellitus, ecological momentary assessment, ambulatory assessment, intensive longitudinal assessment, physical activity, self-management behavior, functioning, social support

## Abstract

**Background:**

Diabetes impacts nearly 25% of veterans. Many veterans do not engage in recommended physical activity and other diabetes self-management behaviors. Type 2 diabetes is generally asymptomatic; as such, the long-term consequences of inadequate self-management and benefits of consistent self-management are not salient in the short term. Furthermore, self-management behaviors typically take place outside of medical visits; however, self-management–related factors are only assessed during medical visits, likely missing large amounts of variability. Thus, ambulatory assessment methods such as ecological momentary assessment (EMA), accelerometry, and continuous glucose monitoring are needed to understand the dynamics of daily self-management and identify potential intervention targets.

**Objective:**

The overarching goal of this study is to understand daily, time-varying factors (comorbid affective symptoms and social context) that influence physical activity, diabetes self-management, glycemic management, daily functioning, and quality of life in participants’ natural environments.

**Methods:**

We are recruiting veterans with type 2 diabetes (target N=100). Participants are required to complete a battery of baseline assessments related to mental health, psychosocial factors, and self-management behaviors. Participants then receive 5 momentary EMA surveys and 1 daily EMA survey per day, in which veterans report comorbid affective symptoms (mood, stress, and pain), social support, social interactions, physical activity, and other self-management behaviors. Momentary surveys are delivered randomly during daily preprogrammed intervals over a 14-day sampling period. Accelerometry and continuous glucose monitoring are also used to assess physical activity and blood glucose, respectively. The first 6 participants also completed interviews assessing their experience in the study and barriers to participation. These test participants informed modifications to the protocol for the remaining participants.

**Results:**

The project received funding in April of 2023. Enrollment began in March of 2023 and is planned to be completed in April 2025. Among the 6 test participants, the overall EMA response rate was 87% (range 74%-95%). The response rate for the EMA survey including daily items (67%, range 21%-93%) was lower than the earlier shorter EMA surveys (89%, range 81%-96%). The mean rate of valid accelerometer wear of at least 20 hours per day was 93% (SD 11%), and continuous glucose monitoring data were available for 91% (SD 17%) of days on average. Participants reported few barriers to completing EMA surveys but noted the random timing of questions made it difficult to plan around, and the end-of-day survey was long. Two participants reported survey items reminded or motivated them to engage in diabetes self-management behaviors.

**Conclusions:**

Assessment tools developed from this study can inform clinical decision-making by considering barriers to self-management that occur in daily life. Clinical applications include tailored, adaptive technology–supported interventions to improve self-management that provide the right type and amount of support at the right time by adapting to an individual’s changing internal and contextual state.

**International Registered Report Identifier (IRRID):**

DERR1-10.2196/53874

## Introduction

### Background

Almost 1 in 4 veterans have type 2 diabetes (T2D), which is over double the prevalence of the general population [1]. Engaging in regular diabetes self-management behaviors, including physical activity, medication taking, following dietary recommendations, and monitoring blood glucose, greatly improves diabetes outcomes [2], but many veterans with T2D do not consistently engage in these behaviors [3]. A more nuanced understanding of veteran-specific barriers to daily engagement in self-management behaviors is needed to develop personalized and impactful interventions to improve self-management.

This study is informed by temporal self-regulation theory, a framework for explaining individuals’ health behaviors, which accounts for temporal dynamics, context (eg, social contact, mood, and pain), and individual factors (eg, beliefs and executive functioning) on health behaviors [4]. Specifically, one of the temporal self-regulation theories posits that engagement in health behaviors is more contingent on immediate outcomes (eg, convenience and discomfort) and less contingent on long-term outcomes (eg, improved health status) and that social, internal, and environmental contexts can influence these temporal contingencies. For individuals with T2D, the long-term consequences of suboptimal self-management may not be immediately salient, as suboptimally managed T2D is often asymptomatic but can lead to several long-term complications [5]. However, other daily experiences, including comorbid affective symptoms and social contextual factors, may have a stronger proximal impact on diabetes self-management behaviors. Comorbid conditions with affective symptoms such as depression, anxiety, stress, and pain are common among veterans with T2D [6-8] and may influence their engagement in self-management behavior. Additionally, veterans’ social context, especially social support, plays a crucial role in daily diabetes self-management [9]. Research suggests that high social support is associated with engagement in diabetes self-management behaviors, which contribute to improved glucose levels, mood, and quality of life [10], while low social support is linked to depression symptoms and functional disability [11], all of which may impede engagement in diabetes self-management behaviors.

Prior research on such influences on diabetes self-management behaviors has mostly relied on cross-sectional studies, which examine static between-person relationships. Few studies have explored within-person variability over time, specifically the moment-to-moment associations between comorbid affective symptoms, psychological states, social support, and daily engagement in diabetes self-management. Intensive longitudinal studies are needed to capture and model fluctuations over short periods of time, context-specific fluctuations, person-specific patterns in behavior [12], interactions between processes at different time scales [13], and both concurrent and lagged temporal dynamics. This study uses ecological momentary assessment (EMA), a real-world ambulatory assessment method that allows for the assessment of mood, behavior, and contextual factors in participants’ own physical and social environments combined with accelerometry and continuous glucose monitoring to continuously assess physical activity behavior and blood glucose levels, respectively. These intensive longitudinal methods can provide valuable insights into these dynamics and help tailor interventions to the specific needs of veterans.

### Aims

**Figure 1:**
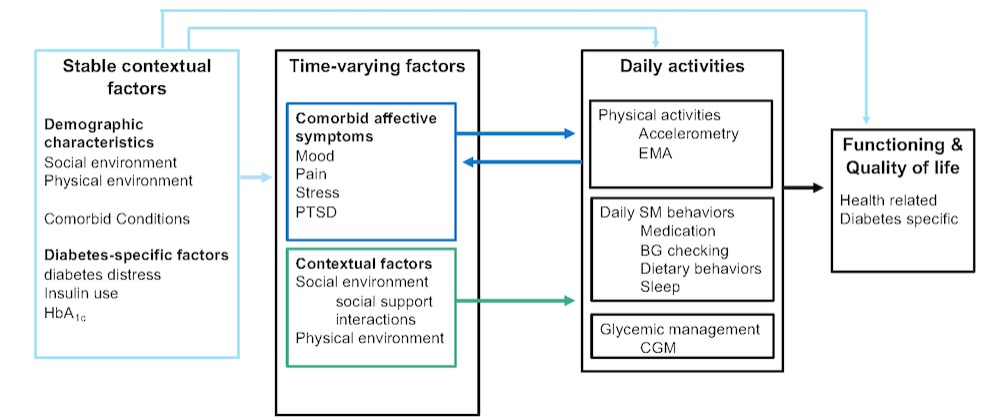
Conceptual model. CGM: continuous glucose monitor; EMA: ecological momentary assessment; HbA_1c_: glycated hemoglobin; PTSD: posttraumatic stress disorder; BG: background.

The overarching goal of this study is to understand daily, time-varying factors (comorbid affective symptoms and social context) that are particularly relevant to veterans and that influence physical activity, diabetes self-management, daily functioning, and quality of life (Figure 1). The specific aims of the study are to (1) use EMA to examine how daily and within-day fluctuations in comorbid affective symptoms influence engagement in physical activity, other diabetes self-management behaviors, and glycemic management; (2) examine social support as it relates to physical activity, diabetes self-management, and glycemic management; (3) capitalize on the advantages of intensive longitudinal data by exploring relationships among other within-person time-varying factors, social contextual factors, between-person demographic and T2D-specific characteristics, and T2D self-management behaviors that may impact daily functioning and quality of life; and (4) leverage intensive longitudinal data to explore temporal relationships among study variables using network analysis. We hypothesize that (1a) greater comorbid affective symptoms will be associated with less concurrent and future engagement in physical activity, diabetes self-management behaviors, and glycemic management; (1b) time-varying within-person factors will predict physical activity, diabetes self-management behavior, and glycemic management even after accounting for stable between-person factors; and (2) daily social support and social interactions will each independently attenuate the association between daily comorbid affective symptoms and subsequent physical activity, diabetes self-management behaviors, and glycemic management. Aims 3 and 4 are exploratory.

## Methods

### Overview of Study Design

Participants use a smartphone app to report comorbid affective symptoms (mood, stress, and pain), social support, social interactions, physical activity, and other self-management behaviors, multiple times per day randomly, during preprogrammed intervals in their natural environment over 14 days. Participants also wear accelerometers and continuous glucose monitors (CGMs) to passively assess physical activity and glycemic management, respectively. The first 6 participants were interviewed about their experience in the study and barriers to responding to EMA items and wearing study devices as well as their understanding of the EMA items. Interview responses in combination with the data collected were used to refine study procedures for the remainder of the participants.

### Target Population and Eligibility

Participants are included if they are users of the VA health care system, diagnosed with T2D, older than 18 years, ambulatory with or without a cane, able to stand without a cane, able to read and understand English, and able to provide informed consent. Participants are excluded if they do not meet the aforementioned criteria or if they are diagnosed with serious or unstable psychiatric illness (ie, unmanaged psychosis, manic episode, or substance use within the past 6 months), psychosocial instability (eg, homelessness) that could compromise study participation, serious or unstable medical illness (eg, dialysis and terminal illness), active suicidal ideation or history of suicide attempt within the past 3 years, concurrent and ongoing engagement in treatment specifically targeting diabetes management, self-reported illness or conditions that would impair the cooperation with the study team or the ability to complete the study, use of a walker, wheelchair, or other assistive devices that will interfere with accelerometry or a score of < 22 on the Montreal Cognitive Assessment (MoCA) or <18 on the MoCA blind [14].

### Recruitment

Participants are recruited using flyers and advertisements posted in the local VA hospital common areas and in clinic spaces likely to serve individuals meeting the eligibility criteria. Additionally, targeted recruitment letters are sent to potentially eligible veterans inviting them to participate in the study, and participants are recruited from lists of veterans who participated in other research studies and expressed interest in being contacted for future studies.

### Ethical Considerations

All participants provide informed consent, and the protocol was approved by the VA San Diego Health Care System institutional review board and R&D Committee (protocol 1227858). Data are deidentified, and key linking participant identifying information to data is kept on a secure server that is only accessible by the research team. Participants receive up to US $200 compensation for participation in the study based on EMA survey response rates and accelerometer wear rates. Specifically, participants receive US $15 for completing the baseline assessment, US $15 for returning devices at follow-up, US $2 for each end-of-day survey, and US $1 for each in-the-moment survey. Participants also receive an additional US $15 for completing 75% (52/70) of EMA questions, an additional US $20 for completing 100% (70/70) of EMA questions, an additional US $15 for wearing their accelerometer for at least 20 hours a day for 75% (10/14) of days, and an additional US $22 for wearing their accelerometer at least 20 hours a day for 100% (14/14) of days. Finally, participants receive a copy of their blood glucose data report collected from the CGMs worn during the study.

### Procedures

**Figure 2:**
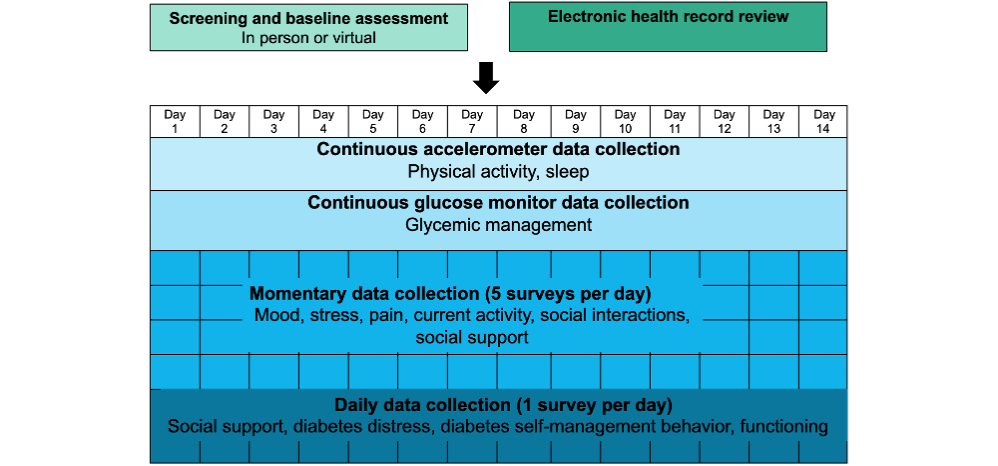
Overview of data collection procedures.

An overview of data collection procedures is shown in Figure 2.

#### Prescreening

Initial screening for interest and eligibility takes place in person or via telephone by the study coordinator. Participants are given an overview of the study and, after providing verbal consent, are screened with a questionnaire covering the inclusion and exclusion criteria. The study coordinator also reviews the veteran’s electronic health record for eligibility criteria. Veterans who are not excluded based on the initial screen and who express interest in participating in the study are invited for continued evaluation of eligibility and the baseline assessment either in person or via a video call.

#### Baseline Assessment Visit

Participants provide written informed consent and Health Insurance Portability and Accountability Act (HIPAA) authorization, followed by the Mini International Neuropsychiatric Interview [15] and the battery of baseline assessments. Participants download the *RealLifeExp* app (LifeData, LLC) to their phones or are provided with EMA devices with the app already downloaded. Participants are trained in the EMA procedures, including how to operate the application, the meaning of all questions and response choices, the timing of the prompts, and procedures for responding to prompts. Each participant is provided with written information on responding to EMA surveys and contact information for crisis lines and all study staff. We also provide participants with an Actigraph wGT3X-BT (Actigraph, LLC) accelerometer and instructions for use. Participants are asked to wear the accelerometer for 14 consecutive days during the EMA period. Participants are also provided 2 CGMs (G6pro, Dexcom) that each last for 10 days, the first of which is placed by the research coordinator on the participant’s right abdomen. Participants are instructed to place the second CGM on their left abdomen on day 9 of the study and remove the first CGM on day 10. CGM data are blinded to participants. Participants already using a CGM will also be asked to wear the study CGMs. Following the baseline assessment visit, participants practiced with the EMA device and accelerometer at home independently for 24 hours. The study team will be available to help the participants in problem-solving any barriers to participation identified during the 24-hour training period.

#### Ambulatory Assessment Procedures

After the 24-hour training period, participants complete 14 days of EMA data collection in which they respond to a set of questions on comorbid affective symptoms, social context, and daily activities 5 times throughout the day, including 1 longer end-of-day survey that also contains questions pertaining to social support and daily T2D self-management behaviors. Prompts are delivered randomly within 2-3–hour time windows. Participants choose between 3 response window options: early (prompts delivered approximately between 7 AM and 7 PM), standard (prompts delivered approximately between 8 AM and 8 PM), and late (prompts delivered approximately between 10 AM and 10 PM). Research staff contact participants after 24 hours, 7 days, 9 days (day of CGM switch), and on day 14 to check in about any issues. Additionally, research staff continually monitor participants’ response rates to EMA survey questions and accelerometry wear time using remote dashboards.

#### Follow-up Visit

Participants return their accelerometer and CGM sensors to the research team after the 14-day data collection period and complete forms for compensation. The first 6 participants completed brief interviews about their experiences in the study, experiences with the EMA platform, completing EMA items, and wearing the accelerometer and CGM.

### Measures

#### Baseline Assessment

Participants complete standardized measures of stable contextual factors including social support, diabetes-specific factors, mental health symptoms, self-management behaviors, functioning, and quality of life. Participants also complete a 24-hour diet recall interview and physical functioning assessment. The full baseline assessment battery is shown in Multimedia Appendix 1 [14-36].

#### Ecological Momentary Assessment

EMA items are shown in Multimedia Appendix 2 [34,37-45]. Items were adapted from existing standardized measures or prior EMA studies when possible. Items were refined based on performance in terms of within-person versus between-person variability and interviews conducted in our pilot study and the initial 6 participants of this study.

#### Actigraphy

Actigraph wGT3X-BT accelerometers assess time spent in light, moderate, and vigorous activities as well as total physical activity counts between each within-day assessment interval and averaged across each day [46]. Total activity counts will be computed for the 60 minutes before and after each prompt, and total activity counts will be computed for each day. Accelerometers will also assess sleep including total sleep time, sleep efficiency, and sleep latency. Accelerometer recordings are time stamped to be combined with EMA data at each prompt. A minimum of 20 valid hours a day will be considered as a valid day for both activity and sleep. A minimum of 10 hours a day will be considered a valid day for activity only.

#### Continuous Glucose Monitoring

Blinded Dexcom G6pro CGMs are used to assess the measures of glycemic management including glycemic management indicator, glycemic variability, percent time in range, percent time in low, percent time in very low, percent time in high, and percent time in very high blood glucose ranges.

### Analytic Plan

#### Protocol Refinement

Data from the initial 6 participants along with their interview responses were used to inform modifications to the protocol. Interview responses were summarized in a table and reviewed with the study team. Response rates for EMA surveys and the number of valid accelerometer and CGM wear days were computed. Intraclass correlations (ICCs) were computed for all EMA items to examine the proportion of the total variance in each EMA survey item attributable to between-person differences versus within-person (ie, day to day) variability.

#### Data Cleaning and Preliminary Analyses

EMA data will be included for all participants completing at least 30% (21/70) of programmed EMA assessments. Additionally, the pattern of missing data will be examined to determine whether missing values are random or systematic omissions. The proportion of participants who complete >80% (56/70) of assessments (including momentary and daily assessments) and the overall dropout rate will be computed. We will examine data completeness using a paired *t* test to evaluate whether participants completed fewer EMA surveys during the second half of the study than during the first half.

We will examine descriptive statistics for all study variables as well as inspect scatterplots subject by subject. Continuous outcome variables will be screened for normality. We will examine bivariate relationships between a set of potential covariates (eg, gender, age, race, comorbid conditions, and diabetes-specific factors) and the primary outcomes. These significant covariates will be included simultaneously in multilevel models (MLMs).

#### Primary Analyses

##### Overview

For aims 1-3, we will estimate MLMs [47]. Models will be estimated with up to 3 levels (prompt, day, and person) with the full-information maximum likelihood method, the between-within method for the denominator degrees of freedom option, and the unstructured variance-covariance matrix for the random effects and residuals. For aim 4, we will use network analysis.

##### Aim 1: Use EMA to Examine How Daily and Within-Day Fluctuations in Comorbid Affective Symptoms Influence Engagement in Physical Activity, Other Diabetes Self-Management Behaviors, and Glycemic Management

To examine the concurrent association between within-day comorbid affective symptoms and engagement in physical activities, we will use MLM to test whether comorbid affective symptoms (depressed mood, stress, and pain) are associated with concurrent (ie, from the same prompt) engagement in physical activity. Physical activity will be assessed using both EMA-reported engagement in physical activity at each prompt (EMA survey) and accelerometer-assessed minutes spent performing physical activity at each prompt interval (total activity counts). The within-person (level 1, prompt-level) version of each comorbid affective symptom predictor variable (ie, depressed mood, stress, and pain) will be entered into each model to partition the variance allowing for the interpretation of within-person results. The within-person terms will be centered on the person-mean, or the participant’s mean value of each comorbid symptom predictor across all prompts for that day, and represent the deviation in each prompt’s comorbid affective symptoms as compared to that individual’s mean value. Within-person comorbid affective symptoms averaged across each day will be included in level 2 (day level). To examine the direction of the association between daily comorbid affective symptoms and daily engagement in future physical activities (EMA survey and accelerometry), we will test multilevel autoregressive cross-lagged path models. These cross-lagged models will examine whether EMA measurements of each comorbid symptom will predict time spent in physical activities the following day while controlling for the effects of time spent in physical activities the previous day, and also whether time spent in physical activities predicted subsequent comorbid affective symptoms after controlling for previous symptoms. In both concurrent and cross-lagged models, we will include covariates in level 3 (person level) to control for between-person differences in these characteristics. We will use an analogous approach for other diabetes self-management behaviors (medication taking, blood glucose monitoring, and diet behavior) and glycemic management (time in range and glycemic variability).

##### Aim 2: Examine Social Support as it Relates to Physical Activity, Diabetes Self-Management, and Glycemic Management

Similar to aim 1, we will examine whether social interactions (ie, alone or with someone else; level 1), companionship or emotional social support (level 2) and instrumental or informational social support (level 2) obtained from EMA surveys are each associated with time spent in concurrent and future physical activity (EMA survey and accelerometry) using MLM. We will include covariates in level 3 (person-level) to control for between-person differences.

##### Aim 3: Capitalize on the Advantages of Intensive Longitudinal Data by Exploring Relationships Among Other Within-Person Time-Varying Factors, Social Contextual Factors, Between-Person Demographic and T2D-Specific Characteristics, and T2D Self-Management Behaviors That May Impact Daily Functioning and Quality of Life

We will examine the relationship between the between-person demographics and diabetes-specific characteristics that may impact daily activities and quality of life using the statistical approaches described above. We will examine the relationship between time-varying comorbid affective symptoms (depressed mood, stress, and pain) and social context (social support and social interactions) with other diabetes self-management behaviors including exercise, diet behaviors, blood glucose monitoring, and medication taking from EMA questions modified using the same statistical approaches described above. We will also examine whether social contextual factors moderate the relationships between comorbid affective symptoms and each outcome variable (physical activity, diabetes self-management behaviors, and glycemic management).

##### Aim 4: Leverage Intensive Longitudinal Data to Explore Temporal Relationships Among Study Variables Using Network Analysis

Network analysis will be conducted to visualize, understand, and compare the associations and covariances among time-varying study variables (eg, comorbid affective symptoms and social contextual factors), physical activity, other diabetes self-management behaviors, and glycemic management. We will estimate within-person networks and between-person networks based on variables in our conceptual model [48]. We will estimate and graph network models in which associations between variables included in the network are drawn if they correlate after controlling for all other variables in the network. Networks will be computed based on partial correlation methods [48]. We will create between-person networks by averaging values from each participant for each physical activity variable, each self-management behavior, and each time-varying factor across the 14 (daily) or 70 (momentary) time points. We will create within-person networks by subtracting the mean of each participant’s score across all time points, such that we estimate a network of interrelationships, centered on each person’s average value during the study. This will allow us to test whether deviations from the mean level for a given variable are associated with variations from the mean of other variables. We will also explore whether network patterns differ based on between-person characteristics (eg, sex and insulin use) that could influence the associations between factors in the network.

### Sample Size

To estimate the sample size needed to detect statistically significant effects for the outcomes outlined in aim 1, we used a power program *EMAtools*, a software specifically designed for EMA data, prior research, and estimates of response rates from our pilot data. There is little prior research with similar populations in terms of examining within-person variability of diabetes self-management behaviors and glycemic management. In terms of physical activity, prior research suggests that we should expect medium effect sizes for aim 1 and small-to-medium effect sizes for aim 2 [37,49]. ICCs for physical activity in similar populations range from 0.12 to 0.34 [49,50]. Specifying 14 days of data collection, and 5 momentary prompts per day, showed that with 100 participants, we are adequately powered to detect the expected effect sizes for aim 1 and small-to-medium effect size for any given outcome (ie, aims 2-3) at 80%. Additionally, we are powered to detect small-to-medium effect sizes with response rates as low as 75%. Network analysis (aim 4) is a relatively new field and research on power requirements is ongoing, but prior studies have found that as few as 30 participants are needed for adequate power to detect variability in network structures [51]. Taken together, the target sample size for this study is 100, with a maximum of 7000 EMA survey responses. Conservatively estimating a 75% response rate, this target would produce 5250 completed EMA surveys that can be combined with corresponding accelerometry and CGM data points. Data from this study can be used in a Monte Carlo simulation to more precisely determine the most appropriate sample size for future studies.

## Results

The study was funded by the Department of Veterans Affairs Rehabilitation Research and Development Service for 5 years. Enrollment began in September of 2022 to collect a sample of 6 test participants which informed subsequent modifications to the protocol. Enrollment restarted in March of 2023 and is planned to be completed in April 2025. Results are planned to be analyzed and published in March 2026.

Among the 6 test participants, the overall EMA response rate was 87% (range 74%-95%). The response rate for the EMA survey including daily items (67%, range 21%-93%) was lower than the earlier shorter EMA surveys (89%, range 81%-96%). The mean rate of valid accelerometer wear of at least 20 hours per day was 93% (SD 11%; range 71%-100%), and CGM data were available for 91% (SD 17%) of days on average (range 57-100). Participants generally reported few barriers to completing EMA surveys but noted that the random timing of questions made it difficult to plan around, and the end-of-day survey was long. Two participants reported accelerometers were uncomfortable at times and 1 participant’s second CGM did not collect data. Two participants reported that survey items reminded or motivated them to engage in diabetes self-management behaviors.

## Discussion

### Summary

There are very few EMA studies examining physical activity and other self-management behaviors in T2D, and there are no studies that we are aware of that examine daily objective physical activity, EMA-assessed self-management behaviors, and CGM-assessed glycemic management among veterans with T2D. Furthermore, the combination of accelerometry and EMA provides both objective and subjective assessments of overall physical activity levels while also distinguishing among activity types.

### Methodological Challenges and Decisions

Modifications to the design of this protocol were made based on a prior pilot study and our test participants [52,53]. Based on response rates lower than 80% and participant feedback from our test participants, we reduced the number of questions delivered at each EMA to 1 per construct. Given our interests in within-person variability, we examined ICC values, representing the amount of within-person versus between-person variability of each item, to inform decisions about which items to drop. Items with lower ICC values, representing greater within-person variability, were favored. Participants in our target population vary in terms of occupational and retirement status and thus participants varied in terms of what times during the day were most ideal for having EMA prompts delivered. To balance variability among participants and capture participants’ typical waking hours and response rates, we decided to offer 3 time window options. Participants also requested to know the exact time EMA prompts would be delivered; however, we decided to keep prompt delivery within random time windows to best capture participants’ experiences across the day.

Social desirability and measurement reactivity pose challenges to assessing daily diabetes self-management behaviors. We initially adapted the Summary of Diabetes Self-Care Activities Assessment scale [16] for daily use but found little variability in responses from participants, and found that most participants endorsed engaging in all self-management behaviors. With our initial test participants, we tried including a response option “this survey reminded me to complete *X* behavior” to gauge reactivity bias; however, no participant selected this option. Additionally, participants reported in interviews that they found the assessment helpful to their diabetes management. We added a component to our instructions emphasizing that we are not intending to change their behaviors in this study. We also modified dichotomous rating scales to be continuous (0-100) to capture variability in behaviors and added new dietary items that included representative food groups without labeling the nutritional value of the food to reduce social desirability biases. We also adapted much of our protocol for remote delivery; however, to date, many participants choose to complete baseline assessment in person.

### Significance

Diabetes is a significant and drastically growing problem among veterans [54] and is related to disability, poor quality of life, and substantial health care costs. Most diabetes self-management activities take place outside of medical visits; however, factors related to self-management are only assessed at the time of medical visits, likely missing large amounts of variability. This study targets this problem by examining time-varying influences of daily diabetes self-management behaviors and daily life functioning, in real-time, in veterans’ own environments.

Results will identify barriers and facilitators of diabetes self-management that when intervened may unleash a cascade of improvement through their interrelationships with other factors. For example, if comorbid affective symptoms are important predictors of physical activity or other self-management behaviors, interventions for T2D management that include additional evidence-based strategies for targeting comorbid symptoms, such as pain management, may need to be evaluated. Adaptive just-in-time interventions can be developed to deliver personalized patient-centered feedback in real time. For example, an intervention could deliver a behavioral activation strategy when a veteran reports high levels of depression. Interventions can be designed to include appropriate timing of prevention strategies or the use of supportive services to help break self-sustaining cycles of poor functioning. Furthermore, assessment tools developed from this study could inform clinical decision-making and treatment planning that considers barriers to self-management that occur outside of medical visits.

## Appendix

Multimedia Appendix 1 Summary of baseline assessment measures.

Multimedia Appendix 2 Summary of ecological momentary assessment items.

Multimedia Appendix 3 Peer-review report from the Career Development Program- Panel II, Rehabiliation Research and Development Parent, IRG Office of Research and Development (RRD9).

## Abbreviations

CGM: continuous glucose monitor
EMA: ecological momentary assessment
HIPAA: Health Insurance Portability and Accountability Act
ICC: intraclass correlation
MLM: multilevel model
MoCA: Montreal Cognitive Assessment
T2D: type 2 diabetes

## Data Availability

The data sets generated and analyzed during this study are available from the corresponding author on reasonable request.

## References

[1] Eibner C, Krull H, Brown KM, Cefalu M, Mulcahy AW, Pollard M, Shetty K, Adamson DM, Amaral EFL, Armour P, Beleche T, Bogdan O, Hastings J, Kapinos K, Kress A, Mendelsohn J, Ross R, Rutter CM, Weinick RM, Woods D, Hosek SD, Farmer CM (2016). Current and projected characteristics and unique health care needs of the patient population served by the Department of Veterans Affairs. Rand Health Q.

[2] Powers MA, Bardsley J, Cypress M, Duker P, Funnell MM, Fischl AH, Maryniuk MD, Siminerio L, Vivian E (2015). Diabetes self-management education and support in type 2 diabetes: a joint position statement of the American Diabetes Association, the American Association of Diabetes Educators, and the Academy of Nutrition and Dietetics. Diabetes Care.

[3] Lynch CP, Strom JL, Egede LE (2010). Variation in quality of care indicators for diabetes in a national sample of veterans and non-veterans. Diabetes Technol Ther.

[4] Hall PA, Fong GT (2007). Temporal self-regulation theory: a model for individual health behavior. Health Psychol Rev.

[5] (2021). National diabetes statistics report: estimates of diabetes and its burden in the United States. Centers for Disease Control and Prevention (CDC).

[6] Banerjea R, Sambamoorthi U, Smelson D, Pogach LM (2007). Chronic illness with complexities: mental illness and substance use among Veteran clinic users with diabetes. Am J Drug Alcohol Abuse.

[7] Shen C, Findley P, Banerjea R, Sambamoorthi U (2010). Depressive disorders among cohorts of women veterans with diabetes, heart disease, and hypertension. J Womens Health (Larchmt).

[8] Trief PM, Ouimette P, Wade M, Shanahan P, Weinstock RS (2006). Post-traumatic stress disorder and diabetes: co-morbidity and outcomes in a male veterans sample. J Behav Med.

[9] Gray KE, Hoerster KD, Reiber GE, Bastian LA, Nelson KM (2019). Multiple domains of social support are associated with diabetes self-management among veterans. Chronic Illn.

[10] Strom JL, Egede LE (2012). The impact of social support on outcomes in adult patients with type 2 diabetes: a systematic review. Curr Diab Rep.

[11] Levy M, Deschênes SS, Burns RJ, Elgendy R, Schmitz N (2019). Trajectories of social support in adults with type 2 diabetes: associations with depressive symptoms and functional disability. Int J Geriatr Psychiatry.

[12] Dunton GF, Rothman AJ, Leventhal AM, Intille SS (2021). How intensive longitudinal data can stimulate advances in health behavior maintenance theories and interventions. Transl Behav Med.

[13] Scholz U (2019). It's time to think about time in health psychology. Appl Psychol Health Well Being.

[14] Nasreddine ZS, Phillips NA, Bédirian V, Charbonneau S, Whitehead V, Collin I, Cummings JL, Chertkow H (2005). The Montreal Cognitive Assessment, MoCA: a brief screening tool for mild cognitive impairment. J Am Geriatr Soc.

[15] Sheehan DV, Lecrubier Y, Sheehan KH, Amorim P, Janavs J, Weiller E, Hergueta T, Baker R, Dunbar GC (1998). The Mini-International Neuropsychiatric Interview (M.I.N.I.): the development and validation of a structured diagnostic psychiatric interview for DSM-IV and ICD-10. J Clin Psychiatry.

[16] Toobert DJ, Hampson SE, Glasgow RE (2000). The summary of diabetes self-care activities measure: results from 7 studies and a revised scale. Diabetes Care.

[17] Fitzgerald JT, Funnell MM, Anderson RM, Nwankwo R, Stansfield RB, Piatt GA (2016). Validation of the revised brief Diabetes Knowledge Test (DKT2). Diabetes Educ.

[18] Kroenke K, Spitzer RL, Williams JB (2001). The PHQ-9: validity of a brief depression severity measure. J Gen Intern Med.

[19] Spitzer RL, Kroenke K, Williams JBW, Löwe B (2006). A brief measure for assessing generalized anxiety disorder: the GAD-7. Arch Intern Med.

[20] Mitchell AM, Crane PA, Kim Y (2008). Perceived stress in survivors of suicide: psychometric properties of the perceived stress scale. Res Nurs Health.

[21] Bovin MJ, Marx BP, Weathers FW, Gallagher MW, Rodriguez P, Schnurr PP, Keane TM (2016). Psychometric properties of the PTSD checklist for diagnostic and statistical manual of mental disorders-fifth edition (PCL-5) in veterans. Psychol Assess.

[22] Polonsky WH, Fisher L, Hessler D, Desai U, King SB, Perez-Nieves M (2022). Toward a more comprehensive understanding of the emotional side of type 2 diabetes: a re-envisioning of the assessment of diabetes distress. J Diabetes Complications.

[23] Amtmann D, Cook KF, Jensen MP, Chen WH, Choi S, Revicki D, Cella D, Rothrock N, Keefe F, Callahan L, Lai JS (2010). Development of a PROMIS item bank to measure pain interference. Pain.

[24] Full KM, Malhotra A, Crist K, Moran K, Kerr J (2019). Assessing psychometric properties of the PROMIS sleep disturbance scale in older adults in independent-living and continuing care retirement communities. Sleep Health.

[25] Ameringer S, Elswick RK, Menzies V, Robins JL, Starkweather A, Walter J, Gentry AE, Jallo N (2016). Psychometric evaluation of the patient-reported outcomes measurement information system fatigue-short form across diverse populations. Nurs Res.

[26] Manapat PD, Edwards MC, MacKinnon DP, Poldrack RA, Marsch LA (2021). A psychometric analysis of the brief self-control scale. Assessment.

[27] Jones CM, Schüz B (2022). Stable and momentary psychosocial correlates of everyday smoking: an application of temporal self-regulation theory. J Behav Med.

[28] Hahn EA, DeWalt DA, Bode RK, Garcia SF, DeVellis RF, Correia H, Cella D (2014). New English and Spanish social health measures will facilitate evaluating health determinants. Health Psychol.

[29] Fitzgerald JT, Davis WK, Connell CM, Hess GE, Funnell MM, Hiss RG (1996). Development and validation of the diabetes care profile. Eval Health Prof.

[30] Sallis JF, Grossman RM, Pinski RB, Patterson TL, Nader PR (1987). The development of scales to measure social support for diet and exercise behaviors. Prev Med.

[31] Crinière L, Lhommet C, Caille A, Giraudeau B, Lecomte P, Couet C, Oppert JM, Jacobi D (2011). Reproducibility and validity of the French version of the long international physical activity questionnaire in patients with type 2 diabetes. J Phys Act Health.

[32] Kirkpatrick SI, Subar AF, Douglass D, Zimmerman TP, Thompson FE, Kahle LL, George SM, Dodd KW, Potischman N (2014). Performance of the automated self-administered 24-hour recall relative to a measure of true intakes and to an interviewer-administered 24-h recall. Am J Clin Nutr.

[33] Morisky DE, Green LW, Levine DM (1986). Concurrent and predictive validity of a self-reported measure of medication adherence. Med Care.

[34] Luo X, George ML, Kakouras I, Edwards CL, Pietrobon R, Richardson W, Hey L (2003). Reliability, validity, and responsiveness of the Short Form 12-item survey (SF-12) in patients with back pain. Spine (Phila Pa 1976).

[35] Burroughs TE, Desikan R, Waterman BM, Gilin D, McGill J (2004). Development and validation of the diabetes quality of life brief clinical inventory. Diabetes Spectrum.

[36] Guralnik JM, Ferrucci L, Simonsick EM, Salive ME, Wallace RB (1995). Lower-extremity function in persons over the age of 70 years as a predictor of subsequent disability. N Engl J Med.

[37] Lüscher J, Kowatsch T, Boateng G, Santhanam P, Bodenmann G, Scholz U (2019). Social support and common dyadic coping in couples' dyadic management of type II diabetes: protocol for an ambulatory assessment application. JMIR Res Protoc.

[38] Moore RC, Kaufmann CN, Rooney AS, Moore DJ, Eyler LT, Granholm E, Woods SP, Swendsen J, Heaton RK, Scott JC, Depp CA (2017). Feasibility and acceptability of ecological momentary assessment of daily functioning among older adults with HIV. Am J Geriatr Psychiatry.

[39] Paolillo EW, Tang B, Depp CA, Rooney AS, Vaida F, Kaufmann CN, Mausbach BT, Moore DJ, Moore RC (2018). Temporal associations between social activity and mood, fatigue, and pain in older adults with HIV: an ecological momentary assessment study. JMIR Ment Health.

[40] Thompson ER (2016). Development and validation of an internationally reliable short-form of the Positive and Negative Affect Schedule (PANAS). J Cross Cult Psychol.

[41] Erwin MC, Dennis PA, Coughlin LN, Calhoun PS, Beckham JC (2019). Examining the relationship between negative affect and posttraumatic stress disorder symptoms among smokers using ecological momentary assessment. J Affect Disord.

[42] Pyatak EA, Hernandez R, Pham LT, Mehdiyeva K, Schneider S, Peters A, Ruelas V, Crandall J, Lee PJ, Jin H, Hoogendoorn CJ, Crespo-Ramos G, Mendez-Rodriguez H, Harmel M, Walker M, Serafin-Dokhan S, Gonzalez JS, Spruijt-Metz D (2021). Function and Emotion in Everyday Life with Type 1 Diabetes (FEEL-T1D): protocol for a fully remote intensive longitudinal study. JMIR Res Protoc.

[43] (2020). ADCES7 self-care behaviors™: healthy eating. Association of Diabetes Care & Education Specialists.

[44] ElSayed NA, Aleppo G, Aroda VR, Bannuru RR, Brown FM, Bruemmer D, Collins BS, Hilliard ME, Isaacs D, Johnson EL, Kahan S, Khunti K, Leon J, Lyons SK, Perry ML, Prahalad P, Pratley RE, Seley JJ, Stanton RC, Young-Hyman D, Gabbay RA (2023). 5. Facilitating positive health behaviors and well-being to improve health outcomes: standards of care in diabetes-2023. Diabetes Care.

[45] Fisher L, Hessler DM, Polonsky WH, Mullan J (2012). When is diabetes distress clinically meaningful?: Establishing cut points for the diabetes distress scale. Diabetes Care.

[46] Santos-Lozano A, Santín-Medeiros F, Cardon G, Torres-Luque G, Bailón R, Bergmeir C, Ruiz JR, Lucia A, Garatachea N (2013). Actigraph GT3X: validation and determination of physical activity intensity cut points. Int J Sports Med.

[47] Bolger N, Laurenceau JP (2013). Intensive Longitudinal Methods: An Introduction to Diary and Experience Sampling Research.

[48] Costantini G, Richetin J, Preti E, Casini E, Epskamp S, Perugini M (2019). Stability and variability of personality networks. A tutorial on recent developments in network psychometrics. Pers Individ Differ.

[49] Maher JP, Dzubur E, Nordgren R, Huh J, Chou CP, Hedeker D, Dunton GF (2019). Do fluctuations in positive affective and physical feeling states predict physical activity and sedentary time?. Psychol Sport Exerc.

[50] Dunton GF, Atienza AA, Castro CM, King AC (2009). Using ecological momentary assessment to examine antecedents and correlates of physical activity bouts in adults age 50+ years: a pilot study. Ann Behav Med.

[51] Lutz W, Schwartz B, Hofmann SG, Fisher AJ, Husen K, Rubel JA (2018). Using network analysis for the prediction of treatment dropout in patients with mood and anxiety disorders: a methodological proof-of-concept study. Sci Rep.

[52] Wooldridge JS, Soriano EC, Harris DE, Afari N (2022). Feasibility and acceptability of ecological momentary assessment of psychosocial factors and self-management behaviors among veterans with type 2 diabetes. Diabetes Spectr.

[53] Wooldridge JS, Afari N (2023). Combining ambulatory assessment methods in veterans with type 2 diabetes: acceptability, feasibility, and lessons learned. https://www.sbm.org/meetings/2023.

[54] Liu Y, Sayam S, Shao X, Wang K, Zheng S, Li Y, Wang L (2017). Prevalence of and trends in diabetes among veterans, United States, 2005-2014. Prev Chronic Dis.

