# Supplementary material for: Daily Functioning of Veterans With Type 2 Diabetes: Protocol for an Ambulatory Assessment Study
Source: JMIR Res Protoc. 2023 Nov 20;12:e53874. doi: 10.2196/53874 (PMC10696502; doi:10.2196/53874)
Supplement: Multimedia Appendix 1 [file resprot_v12i1e53874_app1.pdf]

Variable	Measure	Description
**Screening**		
Psychiatric disorders	Mini International Neuropsychiatric Interview (MINI) Version 7.0[15]	Structured diagnostic interview used for diagnosing DSM-V diagnoses. Exclusionary conditions for the current study included: current psychosis, mania, substance abuse within the 6 months, and suicidality
Cognitive impairment	Montreal Cognitive Assessment (MoCA) or MOCA blind (for remote assessment) [14]	Cognitive screening instrument used to assess cognitive impairment (scores ≤ 23)
**Contextual factors**		
Demographic characteristics	Demographic questionnaire	Age, sex, race, ethnicity, education, and income
Health information	Self-report questionnaire and electronic health record review	Insulin use, medications, medical and mental health diagnoses, HbA1c, time since diabetes diagnosis
Diabetes knowledge	The Revised Brief Diabetes Knowledge Test (DKT2) [17]	14 items assess general diabetes and self-management specific knowledge; 9 additional items about insulin use for respondents using insulin
**Comorbid Affective symptoms and psychosocial factors**		
Depression symptoms	Patient Health Questionnaire-9 (PHQ-9) [18]	9 items assess diagnostic criteria for major depressive disorder
Anxiety symptoms	General Anxiety Disorder-7 (GAD-7) [19]	7 items assess diagnostic criteria for generalized anxiety disorder
Stress	Perceived Stress Scale (PSS-10) [20]	10 items assess the degree to which situations in one's life are appraised as stressful

PTSD symptoms	PTSD Checklist for DSM-5 (PCL-5) [21]	20 items assess DSM-5 diagnostic criteria for PTSD
Diabetes distress	Type 2 Diabetes Distress Assessment System (T2-DDAS) [22]	8 items assess core emotional distress related to diabetes and its management, and 21 items assess the impact of 7 common sources of diabetes distress during the past month.
Pain	PROMIS® pain interference and intensity – short forms [23]	6 items assess the degree to which pain interferes with daily activities over the past week (interference); 3 items assess pain right now, on average, and at its worse over the past week (intensity)
Sleep	PROMIS® Sleep Disturbance-short form [24]	8 items assess severity of sleep disturbance over the past week
Fatigue	PROMIS® Fatigue-short form [25]	4 items assess the experience of fatigue and the interference of fatigue on daily activities over the past week
Self-regulatory capacity	Brief self-control scale (BSCS) [26]	13 items assess dispositional self-control
Motivational beliefs	Adapted from Jones and Schutz [27]	8 items asses expected costs and benefits of engaging in physical activity and diabetes self-management behaviors, expectations for when these costs/benefits will occur and intentions for engaging in these behaviors
**Social environment**		
General social support	PROMIS short form - Informational support 4a - version 2.0, PROMIS short form - Emotional support 4a - version 2.0, PROMIS short form - Instrumental support 4a - version 2.0 [28]	12 items assess information, emotional, and instrumental domains of social support
Diabetes social support	Diabetes Care Profile support scale [29]	19 items assess social support for diabetes from family and friends

Exercise social support	Social Support and Exercise Survey [30]	26 items assess family and friend support around exercise habits
**Daily activities**		
Physical activity	International physical activity questionnaire (IPAQ)– short form [31]	7 items assess work-related, transportation, housework/gardening, leisure-time activity, and time spent sitting. Responses provide information on time and intensity of activity and can be used to calculate Metabolic Equivalent Tasks (METs)
Diabetes self-management	Adapted for daily use from the Summary of Diabetes Self-Care Activities (SDSCA) [16]	11 items assess diabetes self-management behaviors
24-hour diet recall	Automated Self-Administered 24-hour Dietary Recall (ASA24); 2022 Version [32]	Web-based tool that guides respondents through completion of 24-hour diet recall for the previous day. Respondents report foods and drinks by browsing categories and/or searching a list of food and drink terms; allows respondents to add or modify food and drink items at multiple points and assesses food preparation and portion sizes as well as dietary supplement intake. Can be used to calculate an overall diet quality score
Medication taking	Morisky-Green-Levine Medication Assessment Questionnaire (MGL) [33]	4 items assess respondents' taking medications as prescribed
**Functioning/quality of life**		
Health-related quality of life	The Veteran's version of the Medical Outcomes Study 12-item Short Form Health Survey (SF-12) [34]	12 items assess mental and physical health-related quality of life, physical function, and mobility
Diabetes-related quality of life	Diabetes-Quality of life clinical Trial Questionnaire-Revised (DQLCTQ-R) [35]	15 items assess diabetes-related quality of life

Physical functioning	Short Physical Performance Battery (SPPB) [36]	Validated assessment involving a short distance walk, repeated chair stands, and balance tests
