# Supplementary material for: Daily Functioning of Veterans With Type 2 Diabetes: Protocol for an Ambulatory Assessment Study
Source: JMIR Res Protoc. 2023 Nov 20;12:e53874. doi: 10.2196/53874 (PMC10696502; doi:10.2196/53874)
Supplement: Multimedia Appendix 2 [file resprot_v12i1e53874_app2.pdf]

Variable	Measure/Item	Description
**EMA momentary assessments**		
Sleep duration and quality (1st survey only)	"How long did you sleep last night?" (Amount of time)<br>"How rested do you feel?"<br>0 (Not at all rested) to 100 (Extremely rested)	2 items assess sleep quantity and quality
Diabetes management intentions (1st survey only)	"I intend to be physically active for 10+ minutes today."<br>"I intend to take care of my diabetes today."<br>0 (Not at all) to 100 (Absolutely)	2 items assess intentions to engage in health behaviors related to diabetes management
Executive functioning (1st survey only)	"How well have you planned for engaging in physical activity today?"<br>"How well have you planned for managing diabetes today?"<br>0 (Not at all) to 100 (Extremely well)	2 items assess planning for health behaviors related to diabetes management
Physical context	"Where are you right now?"<br>At my home<br>At home of family<br>At home of friends<br>At work<br>At outpatient medical visit<br>In hospital<br>At community center<br>In public business/store<br>In vehicle<br>Outside walking<br>In class/educational setting<br>Inside other<br>Outside other	1 item assesses current physical context (eg, at home, work, in car, outside, etc.) via multiple choice options)

	IF other text box for participant to write-in	
Daily activities	"What are you doing?" [38]<br>IF at home…<br>1. Physical Activity/Exercise<br>2. Preparing food<br>3. Eating or drinking<br>4. Cleaning my home/room<br>5. Laundry<br>6. Budgeting or paying bills<br>7. Showering or grooming<br>8. Changing Clothes<br>9. Watching TV<br>10. Resting<br>11. Social media (eg, facebook, twitter)<br>12. Shopping online<br>13. Internet/computer/tablet use<br>14. Reading/writing/journaling<br>15. Gardening<br>16. Physical labor<br>17. Other physical leisure<br>18. Other non-physical leisure<br>19. Social Interactions<br>20. Working (paid)<br>21. Volunteering<br>22. Schoolwork<br>23. Meditating<br>24. Private religious activities<br>25. Listening to Music<br>26. Smoking<br>27. Arts and crafts<br>28. Playing a musical instrument<br>29. Nothing<br>30. Other	1 item assesses current activity via multiple choice options to activities (eg, watching TV, internet use, instrumental activities of daily living). [38]

	IF not at home<br>1. Physical Activity/Exercise<br>2. Eating or drinking out<br>3. Working (paid)<br>4. Volunteering<br>5. Schoolwork<br>6. Shopping<br>7. Entertainment (cinema, sports, etc.)<br>8. Resting<br>9. Social media<br>10. Internet/computer/tablet use<br>11. Reading, writing, or journaling<br>12. Other non-physical leisure<br>13. Meditating<br>14. Private religious activities<br>15. Meeting (church, AA, etc.)<br>16. Listening to Music<br>17. Smoking<br>18. Riding in a bus, trolley, car or van<br>19. Visiting the beach or park<br>20. Visiting family or friends<br>21. Other physical leisure<br>22. Nothing<br>23. Other	
Social context	"Who are you currently with?" [38,39]<br>"Since the last survey, how many times did you talk with someone else (virtual or in-person) for at least 5 minutes?"<br>Options: 0 (you had no interactions)<br>1 interaction; 2 interactions; 3 interactions; 4 or more interactions	2 items assess current social context and social interactions since the last survey, modified from Paolillo et al. (2018)

Social support (1st survey and end of day survey only)	"Since last night, how emotionally alone did you feel when it came to your diabetes and diabetes management?"<br>"Since last night, how alone did you feel when it came to engaging in physical activity?"<br>0 (not at all alone) to 100 (extremely alone)<br>"Since last night, to what extent did someone help you with diabetes?"<br>"Since last night, to what extent did someone help you be physically active?"<br>0 (Not at all) to 100 (Extremely)	4 items assessing emotional support/companionship and instrumental/information social support for diabetes related health behaviors
Comorbid affective symptoms	Items assessing current mood, stress, pain, and fatigue (eg, "I feel depressed…")<br>0 (Not at all) to 100 (Extremely)	13 items assess mood, depression, anxiety, stress, pain, and fatigue symptoms. Some items modified from the PANAS [40]
Diabetes Distress	"How stressed do you feel about your diabetes or diabetes management right now?"<br>0 (Not at all stressed) to 100 (Extremely stressed)	1 item assessing overall diabetes distress intensity
**EMA daily assessment** (delivered with 5th momentary survey of the day)		
Social support (1st survey and end of day survey only)	"Since this morning, how emotionally alone did you feel when it came to your diabetes and diabetes management?"<br>"Since this morning, to what extent did someone help you with diabetes?"<br>"Since this morning, how alone did you feel when it came to engaging in physical activity?"<br>"Since this morning, to what extent did someone help you be physically active?"	4 items assessing emotional support/companionship and instrumental/information social support for diabetes related health behaviors

PTSD symptoms*	"I feel bothered by reminders of a stressful experience."<br>"I feel avoidant of certain memories, thought, or feelings related to a stressful experience."<br>"I feel bothered by fear, horror, anger, guilt, or shame related to a stressful experience."<br>"I feel overly alert, watchful, or on guard."	4 items assess each PTSD symptom cluster [41]
Daily diabetes-related support (1st and last EMA surveys only)	"Since last night (this morning), how emotionally alone did you feel when it came to your diabetes and diabetes management?"<br>"Since last night (this morning), how emotionally alone did you feel when it came to engaging in physical activity?"<br>0 (Not at all alone) to 100 (Extremely alone)<br>"Since last night (this morning), to what extent did someone help you with your diabetes?"<br>"Since last night (this morning), to what extent did someone help you be physically active?" [37]<br>0 (None at all) to 100 (An extreme amount)	4 items assess participants' interactions with others related to diabetes management
Diabetes self-management behaviors	"Today, how did you do at your overall diabetes self-care?"<br>0 (Very Poor) to 100 (Excellent)	1 item assessing overall diabetes self-management [42]
Diet	"Have you followed a healthful eating plan today?"<br>0 (Not at all) to 100 (Almost always)	2 items assessing overall healthful eating as perceived by the participant as well as specific food types that correspond to

	"Which of the following food types did you eat today?" (Select all that apply)<br><br>Options: Vegetables; Fruit; White fish, chicken, turkey, or eggs; Salmon, tuna, nuts, or avocado; Candy, sweet baked goods, or desserts; Non-diet Soda or fruit juice; Water; Beef, ribs, bacon, or hot dogs; Butter, or ice cream; Chips or fries; Brown rice, oats, or quinoa; White rice, white bread, or flour tortilla	common diabetes nutrition recommendations [43,44]
Blood glucose monitoring	"How well did you do with blood sugar monitoring today?"<br>0 (Very poor) to 100 (Excellent)<br>"How many times did you check your blood sugar today?" (Numerical entry)	2 items assessing overall blood glucose monitoring and number of times checking blood glucose
Medication taking	"How well did you do with taking diabetes medications (pills and/or insulin) as prescribed today?"<br>0 (Very poor) to 100 (Excellent)<br>"For your diabetes medications (pills and/or insulin) today, did you…"<br>(select all that apply)<br>Options: Miss one or more doses; Take at a different time than your doctor recommended; Take a different amount than your doctor recommended; None of these	2 items assessing overall medication taking behaviors
Daily health-related functioning	"Today, how much did your physical health interfere with your daily activities?"<br>"Today, how much did your mental health interfere with your daily activities?" [34]	2 items assess health-related functioning today

Daily diabetes distress	"How satisfied are you with the amount of time it took to manage diabetes today?" "Today, how overwhelmed have you felt with the demands of diabetes?"	2 items assess diabetes-related quality of life today modified for daily use from the DQLCTQ-R [42] and Diabetes Distress Scale [45]
Unexpected events	"What happened today that was out of the ordinary?<br><br>(eg, illness, low blood sugar episode, travel, etc.)"<br><br>This item is available for participants to complete at all times and is an open response format.	1 items to assess whether anything atypical occurred that may impact the responses for that day. Adapted from Pyatak et al. [42]

*Delivered during mid-day survey only.
