# Supplementary material for: Daily Functioning of Veterans With Type 2 Diabetes: Protocol for an Ambulatory Assessment Study
Source: JMIR Res Protoc. 2023 Nov 20;12:e53874. doi: 10.2196/53874 (PMC10696502; doi:10.2196/53874)
Supplement: Multimedia Appendix 3 [file resprot_v12i1e53874_app3.pdf]

SUMMARY STATEMENT

PROGRAM CONTACT: (Privileged Communication) *Release Date:* 09/07/2021

Revised Date:

Application Number: 1 IK2 RX003634-01A2

Principal Investigator *Formerly:* 1IK2RX003634-01A1

WOOLDRIDGE, JENNALEE S

Applicant Organization: VA SAN DIEGO HEALTHCARE SYSTEM

Review Group: RRD9
Career Development Program - Panel II

Meeting Date: 08/11/2021 *RFA/PA:* RX20-006
Council: OCT 2021
Requested Start: 01/01/2022

Project Title: Real-World Assessment of Daily Functioning in Veterans with Type 2 Diabetes

SRG Action: Impact Score:117

Human Subjects: 30-Human subjects involved - Certified, no SRG concerns
Animal Subjects: 10-No live vertebrate animals involved for competing appl.
Gender: 1A-Both genders, scientifically acceptable
Minority: 1A-Minorities and non-minorities, scientifically acceptable
Age: 1A-Children, Adults, Older Adults, scientifically acceptable
Clinical Research - not NIH-defined Phase III Trial

Project Year	Direct Costs Requested
1	211,682
2	215,648
3	220,488
4	224,791
5	229,619
TOTAL	1,102,228

ADMINISTRATIVE BUDGET NOTE: The budget shown is the requested budget and has not been adjusted to reflect any recommendations made by reviewers. If an award is planned, the costs will be calculated by VA Office of Research and Development (ORD) staff based on the recommendations outlined in the BUDGET COMMENT section and any relevant ORD service-specific limitations.

SUMMARY OF DISCUSSION:
A Subcommittee of the Rehabilitation Research and Development Service Scientific Merit Review Board met in Plenary Session and reviewed the above proposal considering all internal and external reviews. This document summarizes the major points of the discussion concerning the proposed project. In any further development of this project, the investigator should consider carefully all the issues reflected in this Summary of Discussion as well as the more detailed comments in the individual critiques.

GENERAL COMMENTS:
The Subcommittee was highly enthusiastic about Dr. Wooldridge as an applicant for this CDA2. It was noted by all that she is a strong applicant, has a clear commitment and career trajectory within the VA, and an exceptional team of mentors with the appropriate expertise for this research project. It is also agreed that it was clearly delineated in the project what each mentor will contribute in terms of mentoring/skills to the application. Dr. Wooldridge was highly responsive to the last critiques strengthening this application. The approach is strong, and likely to generate data for future Merit/RO1 applications, and addresses a significant gap in knowledge. Suggestions for minor concerns (as there were no major concerns) can be found below.

SUGGESTIONS:
- There are numerous measures that should provide a wealth of sufficient data for future grant applications. However, it is suggested that the measures be ordered so the most important is collected first. This should hopefully allow for minimal missing data of the most critical outcomes.
- Clarify what will happen for missing data for lab values that may not be gathered for some individuals (such as HbA1C). The applicant may consider that if this data is critical or will greatly enhance future grant applications that individuals who do not have it in their health records be provided with a lab slip/blood draw to have it drawn.

CONDITIONS:
- The cutoff score for the MoCA should be lowered to a 22 for inclusion. The use of this more conservative cutoff should allow for an expanded recruitment pool while also allowing for exclusion of those who are unable to provide informed consent.
- Please provide a timeline of when the exploratory Aims will be completed.

COMMENTS ON THE BUDGET:
No concerns.

ADMINISTRATIVE NOTE:
The presence of a condition(s) is not an indication that the application has been selected for funding. If a notice of intent to award is issued, then the condition(s) will need to be addressed as part of Just-in-Time (JIT).

DESCRIPTION (provided by applicant):
Type 2 diabetes (T2D) is associated with considerable functional impairment and reduced quality of life. Consistent daily engagement in self-management behaviors, such as physical activity, medication adherence, and monitoring blood glucose is required to maintain daily functioning, quality of life, and glucose control. However, most Veterans do not meet daily self-management targets, particularly physical activity. This is problematic as physical activity is a cornerstone of T2D self-management and represents a fundamental component of functioning. Self-management occurs in the context of patients' own environments. Thus, assessments at healthcare visits likely miss significant amounts of variability in self-management behaviors as well as daily fluctuations in comorbid symptoms and social contextual

factors that influence self-management. The conceptual model guiding the proposal is based on temporal self-regulation theory and posits that because T2D is generally asymptomatic, consequences of poor self-management and benefits of consistent self-management are not salient in the moment. For Veterans with T2D, comorbid symptoms such as mood, pain, and stress are common time-varying momentary barriers to self-management that may particularly impact physical activity. Social context is another well-established set of factors related to self-management among people with T2D. Most research on barriers and facilitators of T2D self-management is cross-sectional and assumes comorbid symptoms and contextual factors are static. Current research does not address the time-varying nature in which these comorbid symptoms and social contextual factors impact physical activity and other self-management behaviors within individuals. Thus, the overarching aim of the current proposal is to understand daily, time-varying factors (comorbid symptoms, social context) that are particularly relevant to Veterans and that influence physical activity, T2D self-management, daily functioning, and quality of life. We will enroll 95 (5 test participants and 90 for the analytic sample) Veterans with T2D and will use ecological momentary assessment (EMA), a method of real-time data collection. Participants will receive 5 momentary EMA surveys and 1 daily EMA survey per day, in which Veterans will report on comorbid symptoms (mood, stress, pain, PTSD), social support, social interactions, physical activity and other self- management behaviors, randomly, during pre-programmed intervals in their natural environment over a 14-day sampling period. Accelerometry will also assess physical activity. With these data, we will address 4 aims: Aim 1) Use EMA to examine within-person time-varying relationships among daily comorbid symptoms (depressed mood, stress, pain, PTSD) and within-day time spent doing physical activities; Aim 2) Use EMA to examine within-person social contextual factors (social support, social interactions) and daily time spent doing physical activities; Exploratory Aim 1) Explore relationships among other within-person time-varying factors, social contextual factors, between-person demographic and T2D specific characteristics, and T2D self-management behaviors that may impact daily functioning and quality of life; and Exploratory Aim 2) Leverage intensive longitudinal data to explore temporal relationships among study variables using network analysis. The successful completion of this CDA will provide the applicant with the necessary training and data to pursue a VA Merit to further examine the complexity of real-world barriers and facilitators of T2D self-management. Results from the current study will help to develop both assessment tools and clinical interventions. Assessment tools developed from the present study can inform clinical decision making that takes into account barriers to self-management that occur outside of medical appointments. Clinical applications include tailored, adaptive technology-supported interventions that provide the right type and amount of support, at the right time by adapting to an individual's changing internal and contextual state.

PUBLIC HEALTH RELEVANCE:
Nearly 25% of Veterans have diabetes, which costs the VA 1.5 billion annually. Diabetes is associated with impaired daily functioning and quality of life. Most Veterans to do not engage in recommended physical activity and other diabetes self-management behaviors. Most self-management takes place outside of medical visits; however, factors related to self-management are only assessed at the time of medical visits, likely missing large amounts of variability. The proposed study will use real-world ambulatory assessment methods to examine daily fluctuations in symptoms like mood, pain, and stress, and social context like social support and daily interactions in relation to physical activities, in real-time, in Veteran's own environments. Results will inform more effective assessment tools and interventions that may improve daily functioning and quality of life. Technology-supported interventions can provide the right type of support at the right time by adapting to an individual's changing internal and contextual state to maximize physical, psychological, and social functioning.

CRITIQUE 1

Applicant: Dr. Wooldridge is currently a VA psychologist completing a Post-Doctoral fellowship in Clinical Research. This is her third submission of the this CDA2. Since her last application she continues to demonstrate commitment to VA research and increased productivity with new publications (all publications currently N=26) as well as additional pilot data. She currently has multiple pending publications including publications from the pilot data presented in this application. Her research focuses on interpersonal influences on physical activity and self-management behaviors in adults with chronic conditions. She has a clear commitment to the VA system, with a "consistent and clear trajectory toward a VA career since graduate school." She demonstrates a well-developed research interest, a history of successful publications and pilot funding. The applicant is clearly at the appropriate stage of her career to apply for the CDA2. She is well-suited for this CDA2 with clear potential to develop as an independent investigator. Further, Dr. Wooldridge has been highly responsive to all comments/critiques from reviewers in the last two applications.

Mentor(s): As in the previous applications, Dr. Wooldridge has assembled an excellent team of 3 mentors and 3 consultants. The mentors and consultants in this application are unchanged. Dr. Niloofar Afari will serve as Dr. Woolridge's primary mentor, together they have published 8 manuscripts.. Dr. Afari is a psychologist, the ACOS for Mental Health at the VASDHS, and Vice Chair of Veterans Affairs and Professor of Psychiatry at UCSD. She has a clear commitment to Dr. Woolridge's career development and a history of successful mentorship. Dr. Afari is an ideal primary mentor for Dr. Woolridge.

Dr. Colin Depp and Scott Roesch will serve as additional mentors with Drs. Genevieve Dunton, Sunder Mudaliar, and Adelaide Fortmann serving as consultants. All the mentors are well funded, with clearly developed research lines and express clear commitment to the applicant. While the mentoring team is large, there is a well-developed plan for mentorship including weekly and bi-weekly meetings with the primary mentoring team, and biannual meetings with all mentors.

Training Program: Dr. Wooldridge has adjusted her recruitment plans to allow time to prepare and submit a Merit in Year 5. Currently enrollment has been adjusted to complete enrollment in the last part of year 4. She currently has a plan in place starting in year 4 to present her Merit idea and receive feedback, and to attend a 2-day grant writing workshop at the start of year 5.

Additionally Dr. Wooldridge proposes that she requires further training in 1) ecological momentary assessment methods and design (done via a class in the first year), 2) statistical analyses for intensive longitudinal data (completed in year 3), 3) technology supported clinical trial development, 4) standards of care for Veterans with type 2 diabetes, and 5) Professional development to transition to an independent VA researcher. The plans for these learning objectives are well developed. She additionally plans to attend a 10-week seminar focused on ethics related to digital health service. She has additionally clarified that UCSD ICTR will also be a part of her training. Overall Dr. Wooldridge has a very well thought out and comprehensive mentoring/training plan.

Scientific Merit:

Significance: This study is well designed and is highly significant with important contributions to the VA and the health care of Veterans with Diabetes. This study proposes to better understand the interplay between comorbid symptoms, social context/support, and daily activity in Veterans with diabetes. The prevalence of diabetes among Veterans is nearly 25%, and the second most common chronic condition among Veterans. Improvement of daily self-management behaviors including diet, medication, and physical activity are necessary to improve overall outcomes for Veterans with type 2 diabetes. By

better understanding this interplay, this study seeks to inform intervention strategies in include appropriate timing of prevention strategies or to maximize the use of supportive strategies to lead to better outcomes for Veterans with T2D.

The use of EMA combined with accelerometry is novel and appropriate to identify the relationships of fluctuations in physical activity and the context of these fluctuations.

Approach: The study proposes EMA to gather real-time assessment in 95 Veterans (5 pilot participants and 90 in the actual sample) 5x/day in which participants respond in their natural environment over a 14-day sampling period. This project is support by solid preliminary data from 3 pilot studies. Dr. Wooldridge has been highly responsive to critiques in previous applications adding additional pilot data. New in this resubmission, she reports preliminary data from Dr. Afari that demonstrates Veterans respond to 3 EMA prompts per day (range 57-100%) while wearing the actigraph for 7 days with an adherence rate of 95% (range 7-100%). This provides evidence that gathering data from the EMA while simultaneously wearing an accelerometer is feasible (this is in direct response to previous questions from reviewers).

Sample: The plan as described is recruiting 95 Veterans to assess mood, stress, pain, PTSD, sleep, social support, social interactions, physical activity, and self-management behaviors 5x/day and one end of day survey. The preliminary data shows the study team were able to quickly recruit and enroll 10 Veterans with T2D for the pilot study, this bodes well for the recruitment of the study. The plan has been updated to start recruitment in the first year and finish by the 4th year.

A clear plan for recruitment including the recruitment of women is provided. The Principal Investigator (PI) and the mentors also clearly address how any individuals with suicidal ideations identified in screening or during the study will be handled. Further this application specifically excludes those with cognitive impairment (screened with the MOCA). In this application it is also specified that engagement in treatment targeting diabetes (MOVE etc.) will be excluded. Inclusion criteria has further been clarified to include that participants are required to stand independently without a cane and complete the short physical performance battery and must be able to ambulate with no more than a cane as an assistive device (they are excluding those who use a walker to allow the use of wrist accelerometers in direct response to questions by previous reviewers).

The applicant expects that ~2-3 individuals per month will be screened, 95 consented and 75 will be necessary to finish the study with adequate power.

Methods: Most endpoints to be measured are clearly described, have strong preliminary data, and are likely to be successfully measured.

Participants will either use their own cell phone, or will be provided with an EMA device which contain the program to deliver EMA surveys. A EMA device will be provided if necessary that follows FIPS-140-2 standards, but that the majority of Veterans in the pilot study had their own device. Training will also be provided to participants with the EMA device prior to use (a strength).

The fourteen continuous days of data collection for EMA and accelerometry is also a strength. The study depends heavily on Veterans wearing the accelerometer and also answering the EMA questions for both Aim 1 and Aim2. Preliminary data demonstrates this is feasible in this population. Further 5 test participants will be included to refine the EMA assessments/accelerometer procedures.

There appears to be a comprehensive plan and outline of measures to be collected at baseline including measures of demographics, diabetes, mood, stress, pain, social environment, physical location, physical activity, and quality of life. New in this application SPPB, PROMIS sleep disturbance, and Fatigue short form were also included. Furthermore, sleep will be measured with the Actiwatch. The number of measures is comprehensive and should allow a wealth of data for future studies.

Data Management: Each participant will be needed at least 4 valid days out of 7 for the accelerometer data to be considered valid. (10 hours per day will be considered a valid day).

Resources: As in the previous applications the facilities are excellent and the applicant appears to be well supported by her mentors.

Protection of Human Subjects: No concerns.

Inclusion of Women, Minorities, and Children: The proposal aims to include both women and minorities. A plan is included to recruitment women specifically for this study. No children will be recruited (outside the proposal's scope).

Critique of Vertebrate Animals Section: Not applicable.

	Yes	No
Research with vertebrate animals?		x

Biohazards and Radioisotopes: Not applicable**.**

Additional Review Criteria (unscored)

Budget (unscored)**:** The budget is adequate for the performance of this project. It includes the PI's salary for an 8/8th VA appointment, and the majority of the rest of the budget is for a study coordinator.

Data Management and Access Plan (for data sharing, unscored)**:** Appropriate.

Overall Strengths:

- As in previous applications the applicant, mentors, and training plans are excellent.
- The applicant shows continued productivity with additional publications, submissions, and pilot grants since the last application.
- The applicant is well suited for a CDA2 with a clear commitment and trajectory to a career in VA research.
- Significant strengths in this reapplication are the additional pilot data, starting recruitment at the end of the first year and completing by the end of the 4th year with a revised time line for a Merit submission.
- The research project itself is significant with the potential to contribute to the mission of RR&D and the VA and also provides ample data for future research studies.
- Ample pilot preliminary data are included.
- The wealth of data gathered is likely to advance multiple future studies even if primary hypothesis are not supported.

Overall Weaknesses: None.

CRITIQUE 2

Applicant: This applicant is very strong, with an exceptional track record in the proposed research. The letters of support are also very strong.

Mentor(s): The mentorship team is comprehensive and has a track record of collaboration and mentorship among its members. It is also noteworthy of how much effort the primary sponsor has committed (12.5%). It is unclear how often the team will meet together with the applicant, but the team's history of collaboration reflects that members are in communication.

Training Program: The training plan is excellent, and very detailed. Moreover, it is clear which mentor will oversee and monitor each set of activities.

Scientific Merit:

Significance: This proposal addresses a significant health problem among Veterans, namely poor self-management of type 2 diabetes with physical activity. This proposal aims to study how within-day and day-to-day fluctuations in co-morbid symptoms (like depression) and social support affect physical activity. This addresses a gap in knowledge by monitoring people throughout the day rather than a single timepoint at a physician visit.

Approach: The approach is strong and well-conceptualized. If successful, it will provide critical data for the applicant's future Merit award. The inclusion of an exit interview for new ideas for a Merit application is a strength. There is a minor but addressable concern regarding the new exclusion criteria of a MoCA score of <26 and its potential impact on recruitment. Given the age range of this sample, this high of a threshold may unnecessarily exclude high numbers of participants. A more conservative cut-off of 21 or 22 may be less stringent, and MoCA score can be used as a covariate if interested. Another minor but addressable concern is the lack of pilot/published data showing how much within-day variability some of the co-morbid symptoms have when assessed with EMA; Figure 4 only shows day-to-day variability, and some evidence of the within-day variability would further justify the proposed aims. Also, the Timeline in Table 1 does not include when the exploratory aims will be accomplished, making it unclear whether there is adequate time to complete them. These are minor concerns and do not detract from the significance and overall merit of the Approach.

Resources: There are sufficient resources for this proposal. However, pilot data suggest that laboratory data (HbA1c) and date of T2D diagnosis may not available for all participants. How will this missing data be handled/acquired?

Protection of Human Subjects: Sufficient.

Inclusion of Women, Minorities, and Children: Sufficient.

Critique of Vertebrate Animals Section:

	Yes	No
Research with vertebrate animals?		X

Biohazards and Radioisotopes: Not applicable.

Additional Review Criteria (unscored)

Budget (unscored)**:** Sufficient for executing proposed research.

Data Management and Access Plan (for data sharing, unscored)**:** Sufficient.

Overall Strengths: This proposal addresses the previous reviewers' concerns, and rigorously addresses an important scientific area for in advancing the health and health care of Veterans. The applicant, mentors, training plan, and environment are strong, and the likelihood of transitioning the applicant to independence for a future MERIT award is high.

Overall Weaknesses: There are no major weaknesses, and only minor concerns in the Approach that do not negatively impact the overall merit of the proposal.

CRITIQUE 3

Applicant:
Jennalee Wooldridge is a post-doc research fellow at the VA San Diego Health Care System and University of California, San Diego. Her Ph.D. is in Clinical Health Psychology and her post-doc is in clinical research.

- 26 publications (9 as first author).
- Dissertation supported by 2 American Psychological Association research awards.
- During post-doc obtained funding from the VA Center of Excellence for Stress and Mental Health to conduct 2 pilot studies to inform her CDA.
- Strong established relationship/track record with proposed mentors.
- Clinical experiences inform research interests.
- Dedication to pursuing a career in the VA.
- career goal is to become a VA investigator with expertise in social contextual influences on diabetes self-management and technology-supported behavioral medicine interventions.
- CDA2 proposal builds on her scientific background in physical activity and health behavior change among individuals with chronic illnesses, particularly T2D.
- Appointment appears dependent on CDA2.
- 2021 – received Society of Behavioral Medicine Diabetes SIG Trainee Excellence award.

The successful completion of this CDA will provide the applicant with the necessary training and data to pursue a VA Merit to further examine the complexity of real-world barriers and facilitators of T2D self-management. Results from the current study will help to develop both assessment tools and clinical interventions.

Mentor(s): 1

- Niloofar Afari, Ph.D. - Primary Mentor – expertise in physical activity, Veteran health, behavioral medicine, technology supported clinical trial development. Weekly meetings.
- Colin Depp, Ph.D. - Co-mentor – expertise in Veteran health, technology supported clinical trial development, EMA methodology, intensive longitudinal analysis – bi-weekly meetings.
- Scott Roesch, Ph.D. - Co-mentor – expertise in behavioral medicine, EMA methodology, intensive longitudinal analysis. Monthly meetings.
- Genevieve Dunton, Ph.D. – Consultant - EMA of physical activity and health behavior, accelerometry, health behavior theory). Quarterly meetings.

- Adelaide Fortmann, Ph.D. – Consultant -psychosocial and behavioral aspects of diabetes, technology-supported interventions, health disparities. Quarterly meetings.
- Sunder Mudaliar – Consultant - diabetes standards of care, recruitment. Quarterly meetings.
- She has a history of successful collaboration with all members of the mentorship team, including Dr. Niloofar Afari (8 manuscripts – 5 first author, 3 under review), Dr. Scott Roesch (2 manuscripts, masters thesis), and Dr. Colin Depp (1 manuscript).
- These mentors and consultants form an expert interdisciplinary team with overlapping and distinct areas of expertise necessary to carry out the proposed project.
- Excellent LOS.

Training Program: The candidate's career goal is to become an independent VA investigator focused on understanding daily processes that influence Veterans' engagement in health behaviors and testing behavioral medicine interventions tailored to the unique needs of Veterans with chronic conditions.

To obtain training in EMA design, statistical analyses for intensive longitudinal data, technology-supported clinical trial development, standards of care and treatment of Veterans with T2D, and overall professional development to transition to an independent VA investigator.
- Training is a combination of coursework and applied learning overseen by mentors. Selected coursework and experiences are thoughtful and directly related to training goals. Specific examples provided.
- Deliverables and benchmarks for success are provided.
- Plans for future Merit award appear appropriate.

Scientific Merit:

Significance:
- T2D is a common and costly problem. Self-management behaviors are needed to maintain daily functioning however most Veterans do not meet self-management targets.
- Studying the within-day and day-to-day variability among study variables.

Approach:
- Good preliminary data demonstrating feasibility and acceptability of approach.
- It appears to be a good bit of data collection, so I worry about participants completing all measures. May want to order the measures so most important are collected first and least important last.
- Network analysis is interesting and innovative.
- Future directions section is well done and insightful.

Resources: Adequate.

Protection of Human Subjects: Adequate.

Inclusion of Women, Minorities, and Children: Adequate.

Critique of Vertebrate Animals Section:

	Yes	No
Research with vertebrate animals?		X

Biohazards and Radioisotopes: No comment.

Additional Review Criteria (unscored)

Budget (unscored)**:** Adequate.

Data Management and Access Plan (for data sharing, unscored)**:** Adequate.

Overall Strengths:
- Responsive to critiques.
- Strong, productive applicant who is dedicated to a career in the VA.
- Progress with preliminary data and manuscripts since last submission.
- Thoughtful research and training path.
- Good trajectory and likelihood for success.
- Strong mentoring team that has a history of working together. All aspects of the research and training covered.
- Well thought out training plan including benchmarks and deliverables.
- Nice conceptual research model.
- Thoughtful research plan.

Overall Weaknesses:
- Appointment appears to be contingent on CDA2 funding.
- It appears to be a good bit of data collection, so this reviewer is worried about participants completing all measures. May want to order the measures so most important are collected first and least important last.

MEETING ROSTER

Career Development Program - Panel II
Rehabilitation Research and Development Parent IRG
Office of Research & Development
RRD9
08/11/2021

CHAIRPERSON(S)

NAYLOR, JENNIFER C, PHD
PSYCHOLOGIST
DURHAM VA MEDICAL CENTER
ASSOCIATE PROFESSOR
DEPT OF PSYCHIATRY
DUKE UNIVERSITY MEDICAL CENTER
DURHAM, NC 27705

OLNEY, CHRISTINE M, PHD
NURSE SCIENTIST
MINNEAPOLIS VA HEALTH CARE SYSTEM
MINNEAPOLIS, MN 55417

PADGETT, LYNNE S, PHD
HEALTH PSYCHOLOGIST
DEPARTMENT OF VETERANS AFFAIRS
VETERANS HEALTH ADMINISTRATION
WASHINGTON DC VA MEDICAL CENTER
WASHINGTON, DC 20422

MEMBERS

ADAMS, RACHEL SAYKO, PHD *
SCIENTIST
INSTITUTE FOR BEHAVIORAL HEALTH
THE HELLER SCHOOL FOR SOCIAL POLICY AND MANAGEMENT
BRANDEIS UNIVERSITY
WALTHAM, MA 02454

ADDISON, ODESSA, PHD, DPT *
RESEARCH HEALTH SCIENTIST
BALTIMORE VA MEDICAL CENTER
ASSISTANT PROFESSOR & DIRECTOR OF RESEARCH AFFAIRS
DEPT OF PHYSICAL THERAPY & REHABILITATION SCIENCE
UNIVERSITY OF MARYLAND, SCHOOL OF MEDICINE
BALTIMORE, MD 21201

ANTONUCCI, SHARON, PHD
DIRECTOR
MOSSREHAB APHASIA CENTER
ELKINS PARK, PA 19027

BORSARI, BRIAN, PHD *
PSYCHOLOGIST, CLINICIAN INVESTIGATOR
SAN FRANCISCO VA MEDICAL CENTER
PROFESSOR
DEPARTMENT OF PSYCHIATRY
UNIVERSITY OF CALIFORNIA, SAN FRANCISCO
SAN FRANCISCO, CA 94121

BRACH, JENNIFER S, PHD, PT
ASSOCIATE DEAN OF FACULTY AFFAIRS AND DEVELOPMENT AND PROFESSOR
SCHOOL OF HEALTH AND REHABILITATION SCIENCES
UNIVERSITY OF PITTSBURGH
PITTSBURGH, PA 15260

BROWN, LILY A, PHD
ASSISTANT PROFESSOR OF PSYCHOLOGY
DIRECTOR, CENTER FOR THE TREATMENT AND STUDY OF ANXIETY
PERELMAN SCHOOL OF MEDICINE
UNIVERSITY OF PENNSYLVANIA
PHILADELPHIA, PA 19104

EVANS, GINA L., PHD
PSYCHOLOGIST
CENTER FOR INNOVATIONS IN QUALITY, EFFECTIVENESS AND SAFETY MENTAL HEALTH CARE
MICHAEL F. DEBAKEY VA MEDICAL CENTER
ASSOCIATE PROFESSOR, BAYLOR COLLEGE OF MEDICINE
HOUSTON, TX 77030

FAIRCHILD, JENNIFER KACI, PHD *
ASSOCIATE DIRECTOR AND FELLOWSHIP TRAINING DIRECTOR
VISN 21 MIRECC - VA PALO ALTO HEALTHCARE SYSTEM
CLINICAL ASSOCIATE PROFESSOR
DEPARTMENT OF PSYCHIATRY AND BEHAVIORAL SCIENCES
STANFORD UNIVERSITY SCHOOL OF MEDICINE
PALO ALTO, CA 94304

GUGLIUCCI, MARILYN R, PHD *
PROFESSOR & DIRECTOR, GERIATRICS RESEARCH
DIRECTOR, U-EXCEL - OLDER ADULT FITNESS PROGRAM
DIVISION OF GERIATRICS
UNIVERSITY OF NEW ENGLAND
COLLEGE OF OSTEOPATHIC MEDICINE
BIDDEFORD, ME 04005

HILGEMAN, MICHELLE M., PHD *
CLINICAL PSYCHOLOGIST
MENTAL AND BEHAVIORAL HEALTH
TUSCALOOSA VA MEDICAL CENTER
TUSCALOOSA, AL 35404

KUNISAKI, KEN M., MD
STAFF PHYSICIAN, MINNEAPOLIS VA HEALTH CARE SYSTEM
MEDICAL DIRECTOR, COPD CASE MANAGEMENT PROGRAM
ASSOCIATE PROFESSOR OF MEDICINE
UNIVERSITY OF MINNESOTA
MINNEAPOLIS, MN 55417

MONTGOMERY, LATRICE, PHD
RESEARCH ASSOCIATE PROFESSOR
ADDICTION SCIENCES DIVISION
DEPARTMENT OF PSYCHIATRY AND BEHAVIORAL
NEUROSCIENCE
UNIVERSITY OF CINCINNATI COLLEGE OF MEDICINE
CINCINNATI, OH 45229

MORIARTY, HELENE JOY, PHD *
NURSE SCIENTIST, CO-PROGRAM DIRECTOR OF VA
INTERPROFESSIONAL FELLOWSHIP IN PATIENT SAFETY,
PROFESSOR, DIANE L. & ROBERT F. MORITZ JR. ENDOWED
CHAIR IN NURSING RESEARCH
CORPORAL MICHAEL J. CRESCENZ VETERANS AFFAIRS
MEDICAL CENTER, PHILADELPHIA VA OFFICE OF
NURSING SERVICE, NURSING RESEARCH
FIELD ADVISORY COMMITTEE
VILLANOVA, PA 19085

PADALA, KALPANA P. MD *
ASSOCIATE DIRECTOR FOR CLINICAL RESEARCH - GRECC
CENTRAL ARKANSAS VETERANS HEALTHCARE SYSTEM
ASSOCIATE PROFESSOR
UNIVERSITY OF ARKANSAS FOR MEDICAL SCIENCES
LITTLE ROCK, AR 72114

PRIMACK, JENNIFER MARIE, PHD *
RESEARCH CAREER SCIENTIST
PROVIDENCE VA MEDICAL CENTER
ASSISTANT PROFESSOR OF PSYCHIATRY
AND HUMAN BEHAVIOR (RESEARCH)
BROWN UNIVERSITY
PROVIDENCE, RI 02906

RAY, ANDREW DONALD, PHD *
ASSOCIATE PROFESSOR OF ONCOLOGY
DEPARTMENT OF CANCER PREVENTION AND CONTROL
ROSWELL PARK COMPREHENSIVE CANCER CENTER
BUFFALO, NY 14263

SCHAEFER, SYDNEY YOSHIE, PHD *
ASSISTANT PROFESSOR
SCHOOL OF BIOLOGICAL & HEALTH SYSTEMS ENGINEER
ARIZONA STATE UNIVERSITY
TEMPE, AZ 85287-9709

SCHERRER, JEFFREY F., PHD *
PROFESSOR
DEPARTMENT OF FAMILY AND COMMUNITY MEDICINE
RESEARCH DIVISION
ST. LOUIS UNIVERSITY SCHOOL OF MEDICINE
ST. LOUIS, MO 63104

SCHUBERT, MICHAEL C PT, PHD *
PROFESSOR
LABORATORY OF VESTIBULAR NEUROADAPTATION
DEPARTMENT OF OTOLARYNGOLOGY HEAD AND NECK
SURGERY AND PHYSICAL MEDICINE AND REHABILITATION
JOHNS HOPKINS UNIVERSITY SCHOOL OF MEDICINE
BALTIMORE, MD 21287

SMELSON, DAVID A, PHD, PSYD *
DIRECTOR, TRANSLATIONAL MENTAL HEALTH RESEARCH
EDITH NOURSE ROGERS VA MEDICAL CENTER
PROFESSOR
DEPARTMENT OF PSYCHIATRY
UNIVERSITY OF MASSACHUSETTS
WORCESTER, MA 01655

TENG, ELLEN, PHD *
DIRECTOR, PSYCHOLOGY TRAINING
MICHAEL E. DEBAKEY VA MEDICAL CENTER
ASSOCIATE PROFESSOR
DEPARTMENT OF PSYCHIATRY
BAYLOR COLLEGE OF MEDICINE
HOUSTON, TX 77030

TESKE, JENNIFER ANN, PHD
ASSISTANT PROFESSOR, NUTRITIONAL SCIENCES
THE UNIVERSITY OF ARIZONA
TUCSON, AZ 85721

UROSEVIC, SNEZANA, PHD *
STAFF PSYCHOLOGIST
MINNEAPOLIS VA HEALTH CARE SYSTEM
ASSISTANT PROFESSOR
UNIVERSITY OF MINNESOTA
MINNEAPOLIS, MN 55417

WAID-EBBS, JULIA KAY, PHD *
SPEECH PATHOLOGY FELLOW
REHABILITATION RESEARCH AND DEVELOPMENT
BRAIN REHABILITATION RESEARCH
CENTER OF EXCELLENCE
MALCOM RANDALL VA MEDICAL CENTER
GAINESVILLE, FL 32608

YALCH, MATTHEW PHD *
ASSISTANT PROFESSOR
DEPARTMENT OF CLINICAL PSYCHOLOGY
PALO ALTO UNIVERSITY
PALO ALTO, CA 94304

SCIENTIFIC REVIEW OFFICER

GROER, SHIRLEY, PHD
SCIENTIFIC REVIEW OFFICER
DEPARTMENT OF VETERANS AFFAIRS
OFFICE OF RESEARCH AND DEVELOPMENT
REHABILITATION RESEARCH AND DEVELOPMENT SERVICE
WASHINGTON, DC 20420

* Temporary Member. For grant applications, temporary members may participate in the entire meeting or may review only selected applications as needed.

Consultants are required to absent themselves from the room during the review of any application if their presence would constitute or appear to constitute a conflict of interest.
